# Predicting Internet Use and Digital Competence Among Older Adults Using Performance Tests of Visual, Physical, and Cognitive Functioning: Longitudinal Population-Based Study

**DOI:** 10.2196/42287

**Published:** 2023-05-05

**Authors:** Tarja Heponiemi, Emma Kainiemi, Lotta Virtanen, Petra Saukkonen, Päivi Sainio, Päivikki Koponen, Seppo Koskinen

**Affiliations:** 1 Finnish Institute for Health and Welfare Helsinki Finland

**Keywords:** internet services, digital exclusion, digital skills, older adults, physical and cognitive decline, mobile phone

## Abstract

**Background:**

The rapidly increasing role of the internet in obtaining basic services poses challenges, especially for older adults’ capabilities of getting the services they need. Research on the predictors of older adults’ internet use and digital competence is especially relevant given that people are living longer than before, and the age profile of many societies is changing rapidly.

**Objective:**

We aimed to examine the associations of objective measures of physical and cognitive impairment with the nonuse of the internet for services and low digital competence among older adults.

**Methods:**

A longitudinal population-based design was used that combined data from performance tests and self-rated questionnaires. Data were gathered in 2017 and 2020 among 1426 older adults aged between 70 and 100 years in Finland. Logistic regression analyses were used to examine the associations.

**Results:**

Those who had poor near (odds ratio [OR] 1.90, 95% CI 1.36-2.66) or distant vision (OR 1.81, 95% CI 1.21-2.71), restricted or failed abduction of upper arms (OR 1.81, 95% CI 1.28-2.85), and poor results from the word list memory (OR 3.77, 95% CI 2.65-5.36) or word list delayed recall (OR 2.12, 95% CI 1.48-3.02) tests had greater odds for nonuse of the internet for services than their counterparts. Moreover, those who had poor near (OR 2.18, 95% CI 1.57-3.02) or distant vision (OR 2.14, 95% CI 1.43-3.19), poor results from the chair stand test (OR 1.57, 95% CI 1.06-2.31), restricted or failed abduction of upper arms (OR 1.74, 95% CI 1.10-2.76), and poor results from the word list memory (OR 3.41, 95% CI 2.32-5.03) or word list delayed recall (OR 2.05, 95% CI 1.39-3.04) tests had greater odds of low digital competence than their counterparts.

**Conclusions:**

According to our results, older adults’ impaired physical and cognitive functioning may hamper their possibilities of accessing internet services such as digital health care services. Our results should be considered when planning digital health care services intended to be used by older adults; that is, digital solutions should also be suitable for older adults with impairments. Furthermore, face-to-face services should be provided for those who cannot use digital services, even if they are assisted properly.

## Introduction

### Background

The digitalization of services has increased rapidly, and the COVID-19 pandemic has given a prominent boost to this phenomenon [1,2]. The possibility of using the internet for services and avoiding face-to-face encounters has offered many benefits during the pandemic, especially for older adults with an increased risk of severe health outcomes caused by a COVID-19 infection [3]. Even before the pandemic, more societal services have been provided on the web, increasing the need to use the internet for accessing services. Digital services provide opportunities for accessing information, communication, and engagement in different leisure activities [4]. In addition, health services are increasingly being delivered digitally [5]. The need to use the internet for important services poses a substantial risk, as it can reinforce existing social inequalities and deepen digital exclusion between those who are able to use the internet and those who are not [6]. Thus, the high delivery of services on the internet could especially impede the ability of some older adults from running their daily errands and taking care of their health and well-being.

Previous survey studies show that older adults have lower access to internet services in Finland [7] and use fewer information technologies in Germany [8] than younger adults. Moreover, a study that combined insurance claims encounters with patient-reported data in the United States showed that older adults used fewer telehealth services than younger adults [9]. Correspondingly, a study from the United States combining data from patients’ electronic health records and surveys showed that patients in the 60 to 69 age group used inpatient portals less than patients in the 18-29 age group [10]. In the United States and Germany, it has been shown that older adults are also less likely to search for health-related information on the internet [11,12].

Digital competence is a crucial prerequisite for being able to use the internet and the required technology. Previous studies have shown that older adults have lower digital competence compared with younger adults [7,13]. In the European Union, 80% of 44- to 54-year-olds estimated that they have the required digital competence in their daily lives, whereas the percentage for those aged >65 years was 44 [14]. For example, a previous survey study from Finland showed that older adults had lower digital competence than younger adults, which also partly explained that they perceived to receive fewer benefits from internet services [7]. This may have a high impact on older adults’ reluctance to use digital services [15]. Instead, in Finland, higher digital competence has been found to postpone the age-related decrease in the use of web-based health and social care services only up to the age of 80 years [13].

Thus, previous studies show that older adults use the internet less often and have less competence in using it. There may be many reasons behind the lower use of the internet among older adults [7,13]. For example, factors such as low access, low education, poverty, poor health, a limited social network, and negative attitudes have been associated with digital exclusion [7,12,14,16]. Moreover, many factors related to older age and aging may pose challenges in using the internet and consequently in gaining and maintaining digital competence. For example, physical and cognitive decline may affect the use of digital services among older adults [17]. In addition, the ability to learn new things often decreases along with growing age [18], and older adults have been reported to be less likely to learn how to use computer-mediated information technologies compared with younger adults [19].

Vision problems, which often occur when people age [20], may restrict certain activities that require good vision and may therefore lead to lower internet use and poor digital competence. A previous case-control study from the United States found that older adults with vision impairment were less likely to use the internet and health information technology compared with those without vision impairment [21]. Moreover, older adults in the United States with vision impairment have been found to use less mobile information and communication technologies (ICTs) than their counterparts in a survey study [22]. An interview study has shown that visually impaired older adults experience interface design and the costs of assistive devices as barriers to internet use [23]. Moreover, people with visual impairment find it difficult to navigate web forms and gain an overview of a web page [24].

Limitations in physical functioning have been identified as important constraints for older adults’ internet use [19]. Physically frail older adults have been found to use less mobile ICT and the internet as well as to have more negative opinions on the usefulness and usability of mobile ICT than older adults who are not frail [25]. Moreover, those older adults who have physical impairments or need assistance with basic daily activities have been found to use the internet less and communicate less using email or SMS text messages compared with their counterparts [22].

Cognitive problems may affect the use of the internet for accessing services, especially among the oldest adults [17]. Previous studies constantly show that good cognitive functioning and high computer use are interrelated [22,26-28]. It has been found that overall cognitive functioning, processing speed, short-term memory, and executive functioning are better among older adults who use the internet on a daily basis compared with those who do not use it daily [29]. Similarly, cognitive functioning, measured as self-rated inductive reasoning and psychomotor speed, has been associated with the basic ability to use a computer [30].

Thus, previous studies suggest that physical and cognitive decline may predispose older adults to lower internet use and consequently to digital exclusion. However, most previous studies have relied on self-ratings to measure physical and cognitive decline. More research about the predictors of digital exclusion, including objective data on physical and cognitive functioning among older adults, is needed. Moreover, it has been emphasized that the association of health and well-being indicators with digital exclusion needs to be examined more thoroughly, especially with longitudinal data [31]. In addition, research examining the associations of physical and cognitive decline with digital competence is still very scarce.

Research on the predictors of older adults’ internet use and digital competence is especially relevant given that Europeans are living longer than ever before, and the age profile of society is changing rapidly. These developments are likely to have profound implications not only for individuals but also for national governments and civil society [32]. Digitalization has been considered to alleviate the challenges posed by demographic change. New types of digital services aim to improve the well-being of older adults and increase the efficient functioning of the service system [33].

### This Study

In the light of these research needs, this study aimed to examine the associations of physical and cognitive impairment indicators measured by performance tests with (1) nonuse of the internet for services and (2) low digital competence in approximately 3-year longitudinal data among older adults aged between 70 and 100 years. More specifically, as indicators of physical functioning, we used results from health examination and performance tests related to near and distant vision, chair stand, hand grip, and abduction of the upper arms. We used the word list memory test and word list delayed recall test as indicators of cognitive functioning. We used longitudinal population-based survey data from 2017 to 2020, also including those older adults who lived in residential homes. We offered a possibility to answer by postal or web-based questionnaire or by telephone for those who had not responded. This ensured that older adults with difficulties in filling the questionnaire were able to take part as well. The longitudinal approach enabled us to draw conclusions related to temporal precedence, and the performance tests of physical and cognitive functioning provided us with more objective measures of functioning compared with widely used self-rated measures. According to the aforementioned previous studies, we hypothesized that both (1) poor physical functioning and (2) poor cognitive functioning are associated with the nonuse of the internet for services and low digital competence among older adults.

## Methods

### Sample

This study used longitudinal data gathered in two waves in (1) 2017, which included both a questionnaire and a health examination (FinHealth 2017 study), and (2) 2020, which included only a questionnaire (follow-up). The FinHealth 2017 study is a comprehensive nationally representative health examination survey covering several aspects of health and well-being, as measured by questionnaires and clinical measurements [34]. The main aim of the FinHealth 2017 study was to produce reliable and up-to-date information on health, well-being, health behavior, and functional capacity as well as their determinants in the Finnish adult population.

**Figure 1:**
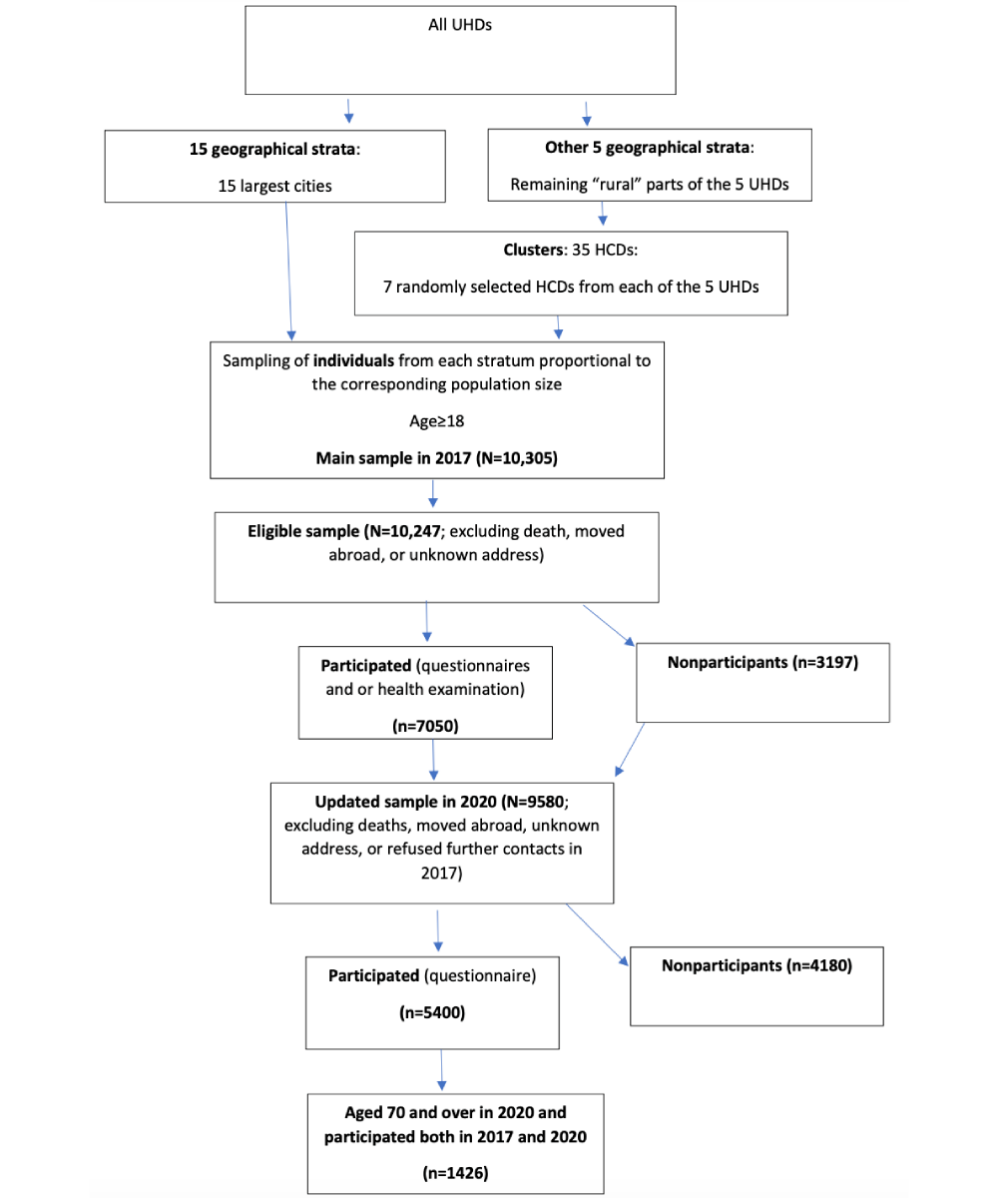
Flowchart of the study sample selection. HCD: health center districts; UHD: university hospital districts.

Figure 1 shows the flowchart of the study sample selection. The FinHealth 2017 study sample, representative of the Finnish adult population, was drawn from the Finnish Population Register, and the data collection was conducted between January and May 2017. The sampling design was a 1- and 2-stage stratified, random sample comprising individuals aged ≥18 years and living in mainland Finland (N=10,305). In addition to community-dwelling people, people living in institutions such as sheltered housing units, care or group homes, or retirement homes were included. A more detailed description of the sample, data gathering, and measures is provided elsewhere [34]. The people were invited to participate in the health examination with an invitation letter, which included a preset appointment time. Moreover, a prenotice postcard was sent 2 weeks before the invitation letter to all sampled persons. It was possible to change the time and place of the appointment to a more convenient one. To achieve the highest possible participation rate, different methods suggested in the European Health Examination Survey protocol [35] were used: reminders, phone calls, and SMS text messages. Altogether, 2733 (participation rate 2733/5079, 53.81%) male and 3219 (participation rate 3219/5168, 62.28%) female individuals participated in the health examination in 2017 [34]. The participation rate was 60.90% (1534/2519) for those who were ≥67 years of age in 2017 and thus belonged to the age range of this study.

For the follow-up survey, conducted during the second wave of the COVID-19 pandemic between October 2020 and January 2021, an invitation letter was sent to the original sample, which was updated to exclude those who had passed away, moved abroad, or refused any further contact (n=9580 for those aged ≥21 years; n=2233 for those aged ≥70 years) [36]. It was possible to fill in the questionnaire on paper or in a web-based format. The questionnaires were available in Finnish, Swedish, and English. The opportunity for a telephone interview was also offered (a shorter version, including key questions for those who had not answered). Reminders were sent by post, SMS text messages, and email for those who had not answered (if their contact information was available). Altogether, 1524 persons aged ≥70 years responded to the follow-up survey (response rate 1524/2233, 68.25%). In the follow-up survey, of those aged ≥70 years, 62% (945/1524) answered on paper, 33% (501/1524) used the web-based format, and 5% (78/1524) answered by a telephonic interview.

This study included only respondents who had participated in the health examination in 2017, responded to the questionnaire in 2020, and were aged at least 70 years during the follow-up data sampling. Thus, this study included 1426 respondents (811/1426, 58.1% female) aged between 70 and 100 (mean 78.2, SE 0.19) years. Those living in sheltered housing were slightly underrepresented in our study, given that 95.4% (1361/1426) of our respondents reported in 2020 that they lived in a regular private residence, whereas this figure was 94.6% in the general population of those aged ≥70 years in Finland in 2020 [37]. An inverse probability weighting (IPW) correction based on variables such as age, sex, marital status, education level, region of residence, language, and possible hospitalizations was used. Previous studies have shown that the IPW method is suitable for adjusting for possible nonresponse bias in the Finnish population [38]. The number of observations varied between 1128 and 1222 in the statistical analyses owing to item nonresponse and a limited number of questions in telephonic interviews.

### Ethics Approval

Participation in the study was completely voluntary, and the participants were provided with an opportunity to withdraw from the study at any time. The FinHealth 2017 study received approval from the Coordinating Ethics Committee at the Hospital District of Helsinki and Uusimaa (reference 37/13/03/00/2016) and the follow-up study from the Ethics Committee II of the Helsinki and Uusimaa Hospital District (HUS/2391/2020).

### Measurements

#### Outcome Variables Measured in the Follow-up in 2020

*Nonuse of the internet for services* was assessed in the follow-up survey in 2020 by asking whether the respondent had used the internet for electronic transactions or services (eg, netbanking, the Social Insurance Institution, tax office, ticket sales, local public services, or web-based shops). The response categories were (1) yes, (2) I need assistance or someone else does it on my behalf, and (3) never. For the analyses, the measure was binary coded as 0=uses the internet independently (deduced from the response category yes) and 1=does not use the internet independently for accessing services (combining response categories never and I need assistance or someone else does it on my behalf).

*Low digital competence* was assessed with a question asking the respondents’ assessment of their digital competence in using web-based services (on a computer or mobile devices). The response options were (1) I do not use them, (2) novice or beginner, (3) I can use the basic services independently, (4) I can use many web-based services effortlessly, and (5) expert (I can teach others). The responses were coded as 0=good or average competence (response options 3, 4, and 5) and 1=low competence (response options 1 and 2).

#### Health Examination Variables From the Baseline in 2017

A detailed explanation of the health examination and measurement methods used can be found elsewhere [34].

#### Visual Acuity

Binocular visual acuity was measured using well-illuminated near and distant vision charts published by Precision Vision [39]. The visual acuity values are presented in Snellen decimals equivalents. Eyeglasses or contact lenses were allowed in the test if the participant normally used them. *Near vision* was examined when the participants held the chart at the distance most appropriate to gain the best acuity. The result was the line with the smallest print on which the participant correctly identified at least 4 out of 5 letters. *Distant vision* was examined when the participant was sitting in a chair at a distance of 4 m from the chart with eyes at the level of the chart. The result was the line with the smallest print on which the participant correctly identified at least 4 out of 5 letters. The measurement of vision acuity was important for this study, given the high prevalence of vision loss among older adults [40]. For the analyses, the results of the vision measures were split into the following tertiles: 1=highest (good vision), 2=average, and 3=lowest (poor vision).

#### Performance Tests of Physical Functioning

The *chair stand test* was conducted using a standard chair with no armrests, with a seat height of 43 to 45 cm from the floor and a seat depth of 39 to 43 cm [41]. First, the participants were asked to sit on the chair and stand up once. If they did not manage to do this or had to use their hands, the test was discontinued. If the participants managed to get up without using their hands, they were asked to get up and sit down 10 times as quickly as possible. A split time was taken at 5 stands, and timekeeping was ended after 10 stands. The test was ended if it was not completed in 120 seconds or if it posed any risk to the participant’s safety. This study used the time of 5 stands because it is a well-standardized and widely used test battery in aging research [41], and poor performance in the 5 chair stand test has been associated with, for example, functional limitations [42] and balance disorders [43]. For the analyses, the results of this measure were split into the following tertiles (separately for male and female participants owing to large differences in their results): 1=highest (good performance), 2=average, and 3=lowest (poor performance). Only participants (n=1187) who completed the test were included in the analyses (some participants refused to do the test or did not manage to perform the test completely or the test was not performed due to contraindications).

*Hand grip strength* was measured using the dominant hand (writing hand) with a Jamar/Saehan dynamometer (Sammong Preston Rolyan 2003). The size of the grip handle was adjusted according to the size of the participant’s hand. The participants sat straight in a chair, feet slightly apart on the floor, and held the dynamometer with the wrist in a neutral position and elbow at 90°. The participant was asked to grip the handle as hard as possible for 3 to 5 seconds. At the same time, the study nurse encouraged the participant to do their best. Hand grip strength measurement is a widely used method in aging research, as handgrip strength has predictive validity for decline in cognition, mobility, functional status, and mortality in older community-dwelling populations [44-46]. For the analyses, the results of this measure were split into the following tertiles (separately for male and female participants owing to large differences in their results): 1=highest (good performance), 2=average, and 3=lowest (poor performance). Only the participants (n=1229) who performed the tests were included in the analyses.

*In the abduction of the upper arms* test, the participants were asked to abduct both arms toward the ceiling. Each arm was rated separately in the following manner: (1) normal, if the arm was raised (near the head, 30° short of the vertical line was accepted); (2) restricted, if the abduction was above the horizontal level but not all the way up; and (3) failed, if the abduction remained below the horizontal level [34]. Shoulder impairment is associated with activities of daily living functions among older adults [47] and is therefore an important component of disability assessments among older adults. The measure was coded as 0=normal (option a) and 1=restricted or failed (options b or c, respectively). If either arm was restricted or failed, the measure was coded as 1 (restricted or failed).

#### Tests for Cognitive Functioning

*Word list memory* was measured by showing the participants 10 words one after another that the participants read aloud and memorized. After this, the participants were given 90 seconds to say the words aloud that they were able to recall. Then, they read the words a second time, in a different order, and this was also repeated a third time. After each round, the participants said the words aloud that they could recall in 90 seconds. The number of words correctly recalled after each of the 3 rounds was summed and split into tertiles as follows: 1=highest (good memory), 2=average, and 3=lowest (poor memory). Word list delayed recall was assessed by asking the participants to repeat the same words approximately 5 minutes later after the grip strength test and the chair stand test were conducted. Word list memory and word list delayed recall are selected tasks in the CERAD (Consortium to Establish a Registry for Alzheimer’s Disease) neuropsychological test battery, originally developed for screening the early phases of dementia and memory disturbances; thus, they are especially relevant to older adults. This measure was coded as 0=good (≥5 words recalled) and 1=poor (<5 words recalled).

#### Demographic Characteristics

Age (in 2020) and sex were obtained from the National Population Register. Age was categorized as 70 to 74.9, 75 to 79.9, 80 to 84.9, and 85 to 100 years.

### Statistical Analysis

Associations of predictor variables with outcome variables were examined using logistic regression analyses. The outcome variables were (1) internet use for services in 2020 and (2) low digital competence in 2020 (in separate analyses). The predictor variables were vision-related variables (near and distant vision), physical performance test variables (chair standing, hand grip strength, and abduction of the upper arms), and cognitive functioning variables (word list memory and delayed recall). All the analyses were adjusted for age and sex. In multivariable analyses, we separately examined the effects of variables related to vision (model A), physical performance tests (model B), and cognitive functioning (model C). Finally, we conducted a fully adjusted model, including all the examined variables (model D). The analyses were conducted in these steps to first examine whether the predictors in each variable group were associated with the outcome variable, omitting the possible effect of variables from other predictor groups, and then in the fully adjusted model D to determine the relative importance of each predictor when adjusted for the effects of all other variables. To avoid multicollinearity, the effects of near and distant vision were examined in separate analyses because of the moderate correlation (*r*=0.44) between these variables. A similar procedure was applied to word list memory and delayed recall (*r*=0.43).

The analyses were conducted using SPSS Statistics (version 27.0; IBM Corp). Methods suitable for weighted data were used, including complex samples logistic regression and complex samples descriptives and frequencies for descriptive statistics.

## Results

### Descriptive Statistics

This study included 1426 respondents aged between 70 and 100 (mean 78.2, SE 0.19) years. Table 1 shows the characteristics of the respondents. More than half of the respondents were female, and the most common age category ranged between 70 and 75 years. More than half of the participants used the internet independently and considered themselves to have high digital competence. The mean near visual acuity was 0.87 and distant visual acuity was 0.98. Of the total participants, 8.7% (124/1426) had a near visual acuity score of ≤0.5, which can be considered as weak vision [48]. Approximately 9 in 10 participants had normal abduction of the upper arms, the mean time for 5 stands was 11.5 (SD 3.60) seconds and the average grip strength was 31 (SD 10.48) kg. Participants remembered on average 19 (SD 4.03) words in the word list memory test, and 18% (257/1426) of the participants recalled <5 words, which has been suggested to indicate having very mild or mild Alzheimer disease [49].

**Table 1:** Characteristics of the participants.

				Values, n (%)		Values, mean (SD)
**From 2020**						
	**Age groups (years; n=1425)**					
		70-74.9	607 (42.6)		N/A^a^	
		75-79.9	382 (26.81)		N/A	
		80-84.9	257 (18.03)		N/A	
		85-100	179 (12.56)		N/A	
	**Sex (n=1426)**					
		Male	615 (43.13)		N/A	
		Female	811 (56.87)		N/A	
	**Internet use for services (n=1373)**					
		Uses independently	782 (56.96)		N/A	
		Does not use independently	591 (43.04)		N/A	
	**Digital competence (n=1372)**					
		Good or average	801 (58.38)		N/A	
		Low	571 (41.62)		N/A	
**From 2017**						
	**Near vision (visus; n=1261)**					0.87 (0.24)
		Highest tertile (good)	578 (45.84)		1.10 (0.12)	
		Average	354 (28.07)		0.80 (0)	
		Lowest tertile (poor)	329 (26.09)		0.57 (0.11)	
	**Distant vision (visus; n=1261)**					0.98 (0.27)
		Highest tertile (good)	324 (25.69)		1.32 (0.16)	
		Average	499 (39.57)		1.00 (0)	
		Lowest tertile (poor)^b^	438 (34.74)		0.69 (0.14)	
	**The 5 chair stand tests (n=1187)**					11.5 (3.60)
		Highest tertile (good)	387 (32.60)		14.9 (4.37)	
		Average	393 (33.11)		10.8 (0.76)	
		Lowest tertile (poor)	407 (34.29)		8.8 (0.87)	
	**Hand grip strength (kg; n=1229)**					30.9 (10.50)
		Highest tertile (good)	434 (35.31)		38.1 (10.08)	
		Average	403 (32.79)		30.1 (7.81)	
		Lowest tertile (poor)	392 (31.90)		23.9 (7.90)	
	**Abduction of the upper arms (n=1226)**					
		Normal	1091 (88.99)		N/A	
		Restricted or failed	135 (11.01)		N/A	
	**Word list memory (n=1214)**					19.4 (4.03)
		Highest tertile (good)	358 (29.49)		23.9 (1.86)	
		Average	480 (39.54)		19.7 (1.46)	
		Lowest tertile (poor)	376 (30.97)		14.9 (2.46)	
	**Word list recall (n=1214)**					
		Good or average (5 or more)	996 (82.04)		N/A	
		Poor (less than 5)	218 (17.96)		N/A	

^a^N/A: not applicable.

^b^Included 1 person who did not see anything.

### Nonuse of the Internet for Services in 2020

The results of the logistic regression analyses regarding internet use for accessing services are presented in Table 2. Near and distant vision were both significantly associated with nonuse of the internet for services in model A, and these associations also remained statistically significant in the fully adjusted model D. Those with poor near vision had 1.9, and those with poor distant vision had 1.8 times greater odds of not using the internet for accessing services compared with those with good vision. Of the physical performance test variables, only the abduction of the upper arms significantly predicted the nonuse of the internet in both model B and the fully adjusted model (model D). Those with impaired abduction of the upper arms had 1.9 times greater odds of not using the internet for services compared with those with normal abduction. Word list immediate and delayed recall were both significantly associated with nonuse of the internet for services in model C, and these associations also remained significant in the fully adjusted model (model D). Those whose word list immediate recall was average had 2 times greater odds and those whose word list immediate recall was poor had 3.8 times greater odds of not using the internet for services compared with those whose word list immediate recall was good. Those who recalled poorly in the delayed recall test had 2.1 times greater odds of not using the internet for services than those who recalled words well.

**Table 2:** Logistic regression for nonuse of the internet for services^a^ (odds ratios [ORs] and their 95% CIs).

		Model A		Model B		Model C			Model D	
		OR (95% CI)	*P* value	OR (95% CI)	*P* value	OR (95% CI)	*P* value	OR (95% CI)		*P* value
**Near vision^b^**			<.001		N/A^c^		N/A			<.001
	Highest tertile (good)	1 (N/A)		N/A		N/A		1 (N/A)		
	Average	1.03 (0.74-1.44)		N/A		N/A		0.95 (0.67-1.33)		
	Lowest tertile (poor)	2.11 (1.53-2.90)		N/A		N/A		1.90 (1.36-2.66)		
**Distant vision^b^**			<.001		N/A		N/A			.004
	Highest tertile (good)	1 (N/A)		N/A		N/A		1 (N/A)		
	Average	1.14 (0.83-1.56)		N/A		N/A		1.10 (0.75-1.62)		
	Lowest tertile (poor)	1.83 (1.31-2.56)		N/A		N/A		1.81 (1.21-2.71)		
**The chair stand test**			N/A		.08		N/A			.14
	Highest tertile (good)	N/A		1 (N/A)		N/A		1 (N/A)		
	Average	N/A		1.32 (0.98-1.77)		N/A		1.33 (0.99-1.80)		
	Lowest tertile (poor)	N/A		1.45 (1.02-2.05)		N/A		1.33 (0.90-1.96)		
**Hand grip strength**			N/A		.87		N/A			.77
	Highest tertile (good)	N/A		1 (N/A)		N/A		1 (N/A)		
	Average	N/A		1.07 (0.80-1.43)		N/A		1.08 (0.80-1.46)		
	Lowest tertile (poor)	N/A		1.08 (0.75-1.54)		N/A		0.96 (0.67-1.39)		
**Abduction of the upper arms**			N/A		<.001		N/A			.002
	Normal	N/A		1 (N/A)		N/A		1 (N/A)		
	Restricted or failed	N/A		2.26 (1.58-3.22)		N/A		1.91 (1.28-2.85)		
**Word list memory^d^**			N/A		N/A		<.001			<.001
	Highest tertile (good)	N/A		N/A		1 (N/A)		1 (N/A)		
	Average	N/A		N/A		2.02 (1.40-2.91)		2.04 (1.41-2.97)		
	Lowest tertile (poor)	N/A		N/A		4.13 (2.94-5.79)		3.77 (2.65-5.36)		
**Word list recall^d^**			N/A		N/A		<.001			<.001
	Good or average	N/A		N/A		1 (N/A)		1 (N/A)		
	Poor	N/A		N/A		2.43 (1.73-3.43)		2.12 (1.48-3.02)		

^a^All analyses were adjusted for age and sex.

^b^To avoid multicollinearity, near vision and distant vision were analyzed in separate analyses.

^c^N/A: not applicable.

^d^To avoid multicollinearity, word list memory and word list recall were analyzed in separate analyses.

### Low Digital Competence in 2020

The results of the logistic regression analyses regarding low digital competence are presented in Table 3. Near and distant vision were both significantly associated with low digital competence in model A, and these associations also remained significant in the fully adjusted model (model D). Those with poor near vision had 2.2 and those with poor distant vision had 2.1 times greater odds of having low digital competence than those with good vision. Of the physical performance test variables, a poorly performed chair stand test and restricted or failed abduction of the upper arms significantly predicted low digital competence in both model B and the fully adjusted model (model D). Those who performed poorly in the chair stand test had 1.6, and those who had restricted or failed abduction of the upper arms had 1.7 times greater odds for low digital competence compared with their counterparts. Word list immediate and delayed recall were both significantly associated with low competence in model C and the fully adjusted model (model D). Those whose word list immediate recall was average had 2 times greater odds and those whose word list immediate recall was poor had 3.4 times greater odds of having low digital competence than those whose word list immediate recall was good. Those who recalled poorly in the delayed recall test had 2.1 times greater odds of having low digital competence than those whose delayed recall was good.

**Table 3:** Logistic regression for low digital competence^a^ (odds ratios [ORs] and their 95% CIs).

		Model A		Model B		Model C		Model D	
		OR (95% CI)	*P* value	OR (95% CI)	*P* value	OR (95% CI)	*P* value	OR (95% CI)	*P* value
**Near vision^b^**			<.001		N/A^c^		N/A		<.001
	Highest tertile (good)	1 (N/A)		N/A		N/A		1 (N/A)	
	Average	1.00 (0.72-1.38)		N/A		N/A		0.91 (0.65-1.28)	
	Lowest tertile (poor)	2.40 (1.76-3.27)		N/A		N/A		2.18 (1.57-3.02)	
**Distant vision^b^**			<.001		N/A		N/A		<.001
	Highest tertile (good)	1 (N/A)		N/A		N/A		1 (N/A)	
	Average	1.23 (0.90-1.70)		N/A		N/A		1.18 (0.82-1.70)	
	Lowest tertile (poor)	2.15 (1.50-3.06)		N/A		N/A		2.14 (1.43-3.19)	
**The chair stand test**			N/A		.02		N/A		.04
	Highest tertile (good)	N/A		1 (N/A)		N/A		1 (N/A)	
	Average	N/A		1.32 (0.97-1.80)		N/A		1.40 (1.01-1.94)	
	Lowest tertile (poor)	N/A		1.68 (1.17-2.41)		N/A		1.57 (1.06-2.31)	
**Hand grip strength**			N/A		.37		N/A		.49
	Highest tertile (good)	N/A		1 (N/A)		N/A		1 (N/A)	
	Average	N/A		0.88 (0.65-1.17)		N/A		0.84 (0.62-1.13)	
	Lowest tertile (poor)	N/A		1.08 (0.77-1.52)		N/A		0.93 (0.66-1.32)	
**Abduction of the upper arms**			N/A		<.001		N/A		.02
	Normal	N/A		1 (N/A)		N/A		1 (N/A)	
	Restricted or failed	N/A		2.05 (1.37-3.07)		N/A		1.74 (1.10-2.76)	
**Word list memory^d^**			N/A		N/A		<.001		<.001
	Highest tertile (good)	N/A		N/A		1 (N/A)		1 (N/A)	
	Average	N/A		N/A		2.03 (1.39-2.97)		1.98 (1.34-2.92)	
	Lowest tertile (poor)	N/A		N/A		3.92 (2.77-5.54)		3.41 (2.32-5.03)	
**Word list recall^d^**			N/A		N/A		<.001		<.001
	Good or average	N/A		N/A		1 (N/A)		1 (N/A)	
	Poor	N/A		N/A		2.45 (1.69-3.53)		2.05 (1.39-3.04)	

^a^All analyses adjusted for age and sex.

^b^To avoid multicollinearity near vision and distant vision were analyzed in separate analyses.

^c^N/A: not applicable.

^d^To avoid multicollinearity, word list memory and word list recall were analyzed separately.

## Discussion

### Principal Findings and Comparison With Prior Research

This study examined the longitudinal effects of physical and cognitive decline measured using performance tests on internet use and digital competence. Our results suggest that physical and cognitive functioning predicts nonuse of the internet for services and low digital competence. More specifically, we found that poor near or distant vision, restricted or failed abduction of the upper arms, and poor results from the word list memory or word list delayed recall tests predicted nonuse of the internet for services. In addition, poor near or distant vision, poor results from the chair stand test, restricted or failed abduction of the upper arms, and poor results from the word list memory or word list delayed recall tests predicted low digital competence.

Our finding that poor near or distant vision measured with performance tests predicts nonuse of the Internet for services and low digital competence is congruent with a previous study showing that self-rated vision impairment has been associated with lower use of the internet and health information technology among older adults [21]. Moreover, older adults with self-rated vision impairments have been found less likely to use technology compared with those without vision impairments [22].

It has been found that older adults with vision impairment face challenges that discourage them from using the internet [23]. The need to rely on memory, difficulties locating links and information, anxiety over security, and mental workload have been highlighted among the main challenges encountered by those with visual impairment when using the internet [24]. The demands their impairment sets on internet services are often not considered, and the layout and color schemes are often found to be confusing [23]. In addition, the complexity of internet services and rapid advancements in technology present a major challenge [23].

According to our findings, physical impairments, such as difficulties in the abduction of the upper arms and poor physical strength of the upper limbs, may endanger older adults’ capabilities of internet use and maintaining good digital competence. Previous cross-sectional studies that measured physical impairment using self-ratings support these findings [22,25]. For example, the physical frailness of older adults has been associated with low use of mobile ICT (such as smartphones and tablets) and the internet [25]. Physical capacity impairment or the need for assistance for basic daily activities has been associated with lower use of the internet and communication through emails and SMS text messages [22]. In a longitudinal data set, it has been shown that deteriorated self-rated health predicts low internet use for accessing services among older adults [27]. Our study confirms these findings using objective measures of physical impairment and longitudinal data.

Our finding that poor performance in word list memory and delayed recall of words predicts nonuse of the internet and low digital competence is congruent with findings showing that impairments in memory are associated with low use of the internet, email, and SMS text messages [22,50]. Good cognitive functioning has been associated with higher computer and internet use [26,28,29] and the basic ability to use a computer [30]. Cognitive impairment has been found to be an important hindrance for the use of personal health records among residents living in subsidized housing projects [51].

Our results show that, in principle, similar factors are associated with both internet use for services and digital competence with the exception of the results of the chair stand test predicting digital competence but not nonuse of the internet for services. This is congruent with previous studies showing that digital competence and internet use are interrelated [7,13].

### Implications

Our results suggest that physical and cognitive decline as well as vision problems predispose older adults to nonuse of the internet for services and low digital competence. Technological improvements would be important in addressing these age-related barriers that affect older adults’ use of technology [52]. Good usability, ease of use, and considering users’ needs when digital services are designed could boost use [53] and improve older adults’ confidence in using the internet [54].

People with disabilities and impairments could benefit greatly from the provision of assistive technology and accessibility tools to help them use computers and the internet [24]. For example, large print keyboards, Braille embossers, Braille displays, voice synthesizers, haptic mice, and spatial feedback devices may help people with vision disabilities [55]. Moreover, multimodal solutions and interfaces might be of help [56]. The inclusion of larger clickable labels in internet services could facilitate the use of services for older adults with a motor disability [57]. Those with cognitive impairments could benefit from simplified textual content, linear navigation, placing important functions in the center of the web page, the ability to press buttons from outside the visual layout, and slow-paced audio assistance [58].

However, the assistive solutions may be complex, ineffective, and expensive to purchase [55]. Moreover, people need the training to use these special assistive equipment [24]. There are also other barriers, including limitations for interpretation by assistive devices and policies that do not support accessible design [22,55].

European Union legislation obligates the providers of internet services to assess and report on the current status of accessibility of their service and to provide an electronic channel for related customer feedback (*the accessibility of the websites and mobile apps of public sector bodies* [59]). However, the legislation has been criticized for facilitating access for people with physical or visual impairment but neglecting the needs of people with cognitive decline [60]. Developing internet services accessible for people with cognitive impairment should be an equal priority, given that memory-related challenges are increasingly being faced by the aging population [61] and given that of all the health examinations in our study, impaired cognitive functioning was most strongly associated with the nonuse of the internet for services.

Certain ﬁle types and web features described above are inaccessible to users navigating the internet through a screen reader.

Additional assistance could provide beneﬁt to the collaboration process when performing web-based tasks.

Digital competence is a prerequisite for internet use, and it could be improved by facilitating access to web-based courses, for example, by providing technical support and family support as well as training [30]. Moreover, active teaching and learning methodologies could be used [30]. Younger people could be educated to mentor older adults [62] or games could be used to develop the skills required to use smart devices [63].

### Strengths and Limitations

The strength of this study is its longitudinal design, which combined data from performance tests and self-rated questionnaires. Therefore, we were able to identify older adults who had objectively measured impairments in physical and cognitive functioning. This enabled us to fill gaps in earlier findings, which have mainly been based on subjective measurements and cross-sectional designs. Moreover, a strength of our study was a national population–based sample that had a fairly good participation rate. We had the possibility of using individual-level register-based data to correct for nonparticipation, which allowed us to generalize our findings to the entire older Finnish population. In addition, the generalizability of our results was improved by offering the possibility to participate by telephone and by including older adults who lived in sheltered housing units, care or group homes, or retirement homes.

Our study had some limitations that should be considered when interpreting our findings. Part of our data is based on self-reported data, which can lead to problems associated with common method variance and inflation of the strengths of the associations. We used IPW correction [38] based on variables such as age, sex, marital status, education level, region of residence, language, and possible hospitalizations to correct possible response bias, but there is no certainty as to how well they correct the bias related to functional limitations. Previous studies have shown that the IPW method is suitable for adjusting for possible nonresponse bias among the Finnish population.

We controlled for age, sex, and examined factors; however, the possibility of residual confounding still exists. For example, we did not include social support; thus, the possibility of getting support in internet use and access through relatives might have affected our results. Moreover, even though our health examination covered many aspects of physical and cognitive functioning, there were factors especially relevant to older adults that our study did not include, such as sociodemographic factors, executive functioning, mobility, and time. For example, participants’ functionality could have deteriorated substantially during the study period, which was not examined in this study. Future studies should examine the effects of these measures in more detail. Moreover, including those who lived in sheltered housing or corresponding arrangements may have confounded our results, given that their profile and pathology may differ from those of other older adults. This should be considered when interpreting the results of this study. In addition, we excluded participants who did not have proper results from the 5 chair stand tests or the hand grip test, which possibly excluded those with the poorest physical functioning. This may have affected our results, and including those with the poorest physical functioning might have given a more significant association between physical functioning and digital use or competence. Moreover, generalizing our findings to other countries that are in a different state of digitalization of services or have disparate digital services should be done with caution, given that Finland is a forerunner in the digitalization of services. In addition, a larger proportion of older adults in Finland use the internet and digital services than older adults in many other European countries [64].

When asked to assess the respondents’ digital competence in using internet services, those who responded to that question by saying that they did not use internet services were coded to the low digital competence group. This can be justified, for example, by the strong connection between digital competence and internet use found in previous studies [7,12]. However, the results regarding digital competence can be seen as preliminary and should be verified in future studies using more detailed and accurate measures of digital competence.

### Conclusions

Our results suggest that older adults’ poor physical and cognitive functioning is associated with nonuse of the internet and low digital competence. The digitalization of health care services has increased unprecedentedly lately and, therefore, this possibly higher risk for digital exclusion among older adults who have functional problems should be kept in mind when planning health care services. Digital solutions should be suitable for older adults with impairments to meet the rapid growth of the aging population and their growing need for services. Furthermore, nondigital services should be provided for those who cannot cope with digital services, even if they are assisted appropriately. Future studies should more thoroughly examine the promoters of digital health service use, as well as the capabilities of older adults with physical and cognitive impairments to learn to use them.

## Abbreviations

CERAD: Consortium to Establish a Registry for Alzheimer’s Disease
ICT: information and communication technology
IPW: inverse probability weighting
OR: odds ratio
